# Informal Carers Training: In-group Social Learning as an Effective Method for Quality Care Empowerment

**DOI:** 10.3389/fsoc.2019.00063

**Published:** 2019-08-30

**Authors:** Jože Ramovš, Ana Ramovš, Ajda Svetelšek

**Affiliations:** Anton Trstenjak Institute of Gerontology and Intergenerational Relations, Ljubljana, Slovenia

**Keywords:** long-term care, informal care, informal carers training, group social learning, group method, family carers, intergenerational solidarity, demographic solutions

## Abstract

In this article, a method of in-group social learning used for informal carers training is presented. This method was developed by Jože Ramovš and his team at the Anton Trstenjak Institute of Gerontology and Intergenerational Relations primary for the fields of holistic health prevention and aging; later on, special attention has been given to its development for training of informal carers as the latter often carry the most significant part of the long-term care burden. In the first part of the article, the need for such a method is discussed through a review of current international demographic and long-term care situations. In the second part, a novel method for the training of informal carers is introduced. Finally, the results of the evaluation analysis of 453 persons who participated in the training are presented and compared with the results of the nationally-representative study. The results show that the method of in-group social learning has a great potential for quality care empowerment of informal carers as well as for holistic development of intergenerational solidarity in the modern age. Furthermore, they open new possibilities for research and present directions for further development and implementation of the described method within this important field.

## The Review of Demographic Change and Long-Term Care in the Twenty-First Century Underlines the Need for Informal Carers Training and In-Group Social Learning Method

Long-term care refers to services designed to support old, disabled or ill people who cannot perform one or more basic daily tasks called Activities of Daily Living (ADL)—getting out of bed, personal hygiene, dressing, functional mobility, self-feeding, taking medicines, excretion and maintaining daily social interactions or Instrumental Activities of Daily Living (IADL)—cooking, cleaning, washing and ironing, shopping, financial transactions, transport and other important tasks. This and other similar definitions constitute a framework for modern European systems of long-term care (Leichsenring et al., 2013) and their tools for assessment of care needs and carers' rights (Richtlinien, 2016).

Throughout mankind's history there have been two crucial anthropological observations about caring for the disabled: (1) contributions of stronger individuals toward weaker (called solidarity) is one of the essential characteristics of the human species; (2) caring for ill, frail or disabled individuals by families, relatives and neighbors (partly even by local, religious or other types of communities) is assumed in traditions of all civilizations.

In the twenty-first century, two essential phenomena have made a significant impact on the field of care for disabled members of the community. The first phenomena are the demographic changes leading to an aging population, which have severe effects on requirements and provision of long-term care. Second is the need for new social models of solidarity between generations, due to the radically changed domestic situations compared to traditional models of coexistence.

European policy aims to respond to demographic changes and to build on the human anthropological tradition of solidarity (European Commission, 2005). Health, social, spatial, infrastructure and gradually also educational experts are responding to these changes cooperatively, through accelerated development of methods aimed to improve intergenerational cooperation, good quality aging, and, importantly, long-term care. Accordingly, the development of our novel method of in-group social learning used for training of informal carers, which will be presented here and is based on the previously mentioned anthropological facts, aims to provide some of the answers to this challenging new demographic situation.

### Demographic Situation and Long-Term Care

In the transition from the Twentieth to the Twenty-first century, the fertility rate in developed countries decreased to below the replacement level of its population, which is 2.1 children/woman. Together with prolongation of life expectancy, continuing from the Twentieth century, and the large “baby boom” generation born after the 2nd World War, this has resulted in accelerated aging of the population.

While traditional societies had ~10% of their populations aged 60+, Europe's share of 60+ people is currently around 25% and will increase up to 35% by 2050. The proportion of people aged 80+ has been increasing even more rapidly and is projected to triple around the world by 2050 (United Nations, 2017a). Considering that majority of people aged 80+ need support with their ADL and that support with IADL is needed from 5 to 10 years earlier, it is no wonder that proportion of European population in need of long-term care services is increasing (European Commission, 2014). The same could be observed in most areas of the United States, Canada, Japan, China, Australia, and other developed countries (United Nations, 2017b).

Innovative solutions in contemporary long-term care can also be transferred to other parts of the world, which, despite maintaining high birth rates, are nevertheless experiencing delayed demographic changes affecting long-term care of old and disabled populations. In countries of Africa, South America and, to a lesser extent, Asia, this phenomenon is a consequence of rapid migration to urban areas, in particular to suburbia of fast-growing cities, and the disintegration of traditional families and their patterns of coexistence, along with the fact that most of these countries do not have advanced systems of social care (e.g., universal pension income, health insurance and a social network of public care services) which could replace the shortfall of traditional care.

Over the past two centuries, industrialization and urbanization led to structural adjustments of care in parts of Europe and North America. Professional care institutions and services were gradually introduced, especially over the last 50 years, starting with the retirement homes, followed by home care and various other services for respite, day, night, social and health care, including contemporary hospice models for palliative care; all these programs are provided by formal carers.

At the end of the Twentieth century and the beginning of the Twenty-first century, it became apparent there was an increased need for care and support of informal carers. The important turning point in Europe was European research project about family care EUROFAMCARE—Services for Supporting Family Carers of Elderly People in Europe: Characteristics, Coverage and Usage (Mestheneos and Triantafillou, 2005), with the participation of six EU countries: Germany, Greece, Italy, Poland, Sweden, and the United Kingdom. Slovenia was partly involved in this project, during which the first Slovenian research on informal care was carried out (Hvalič Touzery, 2007). Since then, informal care has become the focus of many international research studies, including a longitudinal, international project SHARE—Survey of Health, Aging, and Retirement in Europe.

The data mentioned above (Mestheneos and Triantafillou, 2005) showed that informal carers of disabled older persons are predominantly family members or, to a lesser extent, their neighbors, volunteers, and friends. As Stecy Yghemonos, an executive manager of Eurocarers (European Association Working for Carers) pointed out, informal carers represent 20% of the European population (nearly 100 million people) and provide 70–90% of all care in today's Europe (Yghemonos et al., 2018). Needless to say, European long-term systems would not be sustainable without their contribution. However, this crucial resource is under pressure. Many people find themselves placed in the role of carer overnight and face extreme challenges: a lack of skills and knowledge about how to provide care, about the diseases, and about the communication with disabled people; a lack of time for rest; and the inability to cope with their own personal physical and mental health, including sense of helplessness and fear of care receiver's health deterioration (Ramovš, 2013). Current global demographic and socio-economic trends are further compounding this strain, such as lower birth rates, smaller family units, increasing mobility leading to greater physical distances between relatives, the rising number of women entering the labor market and a prolonged working life due to delayed retirement (Eurocarers, 2017). Despite this, informal carers' interests are still rarely considered in European policies in a consistent and across-the-board manner (Eurocarers, 2017).

In Slovenia, a nationally representative field study was carried out among older persons aged 50+ on their needs, potentials, and standpoints; this research also provided in-depth data about care (Ramovš, 2013). According to this data, long-term care in Slovenia is provided by 220,000 informal carers (~11% of Slovenian population) who provide care regularly, from a few hours per week to 24 h per day. Among them, there are ~200,000 family carers (relatives) and ~20,000 other informal carers (neighbors, volunteers, or friends), and they provide care to 75% of persons in need of care. For the remaining quarter of this group, to whom the care is provided in institutions, a coordinated inclusion of relatives and volunteers can also significantly improve their quality of life.

Since a long-term care system has not yet been adopted in Slovenia, informal carers are left unsupported, despite politicians declaring they recognize the need for respite care and training of informal carers. The interdisciplinary strategy of care for older persons published in 2008 titled “*Solidarity, good intergenerational relations and quality aging of the population,”* predicted: “…*for the families who care for an older family member proper training should be provided together with various forms of local support services and day or respite care for the older family members; to support adoption of measures for more flexible working time and to support the right to the shortened working time, without the closing of social security, for the employed person due to the urgent care of the close family relative*.” Republic of Slovenia. Ministry of Labour (2008). However, its execution still waits for the adoption of a modern system for long-term care: the last legislative proposal on long-term care was introduced at the end of 2017 but was later removed.

To conclude, results from studies and practical experiences lead us to three main findings on the demographic situation and long-term care:

1. Without informal care, no universal, humane, societally, and economically sustainable care can be ensured by any professional, public, or commercial system in the present times or the future.
2. For family and other informal carers provision of care is getting increasingly difficult.
3. An answer lies in an integrated national system of long-term care that brings together all sources of care—informal and formal: family and other informal carers, organized professional carers, programs, institutions, public resources and resources of people in need—that is systematically organized on a national level, carried out on a community level and is directed toward home care and deinstitutionalization. This system will incorporate training and support for family and other informal carers. This third finding was recently recognized also by the European Union in the form of the European Pillar of Social Rights on the Long-Term Care (European Parliament, 2017).

The method of in-group social learning, outlined in this article, was developed as a response to the needs of informal carers for quality training. It covers the topics recognized as most pressing for this group and takes into consideration their individual capabilities and limitations. As stated by many sources (e.g., Turner and Street, 1999), quality training is one of the best ways to empower informal carers.

### Need for a New Solidarity

With the disintegration of traditional families and their patterns of coexistence, social capital from millennia-old traditions with traditional patterns of solidarity is expiring. A Green Paper “Confronting demographic change: a new solidarity between the generations” (European Commission, 2005) is a response to this new situation.

Delivering care plays a significant role in developing persons' ability for solidarity. Consequently, in the times of population aging, the great need for care presents not only great burden but also offers a unique opportunity to develop contemporary solidarity. This is especially important as, despite the successful scientific and technical progress during the last two centuries (which consequently improved physical health and life expectancy), the development and understanding of human relations and the person as a whole in Europe lagged behind.

Long-term care has two dimensions (as does any other domestic, volunteer, or professional work with people in need or distress):

1. It is a service or task which responds to the concrete need of a person who is not able to perform this service or task by himself/herself.
2. It is a personal relationship between two human beings.

Services are often performed better, faster, and cheaper with professional skills and technical tools or aids; and indeed, care services are nowadays also available on the market and will likely be largely supported by information and communication technology (ICT) in the future. However, such care services only support single directionality of the communication: from carer to care receiver (user of this service).

On the other hand, the human relationship between the person who needs help to survive and the stronger person who can provide this help, functions as two-way open communication which preserves and enhances the human dignity of both, the care receiver and the carer. This is a quality that cannot be offered on the market or substituted by ICT.

When service or task and human relationship are in equilibrium, they offer a possibility for the development of the above-mentioned new solidarity. The carer in his/her position of strength through care enables the care receiver to live and to experience a better quality of life. On the other hand, when asked about benefits in a comprehensive cost-benefit analysis of care (Ramovš et al., 2013), carers listed the awareness of successfully fulfilled obligation toward their family members as important, they noted the expressed gratitude of care receiver and they expressed hope to receive help from others in the future, if needed. This and other parts of the study show that, while giving help to disabled or aged persons, a majority of carers experienced the development of empathy, compassion for other human beings and solidarity—human abilities that are key to any quality interpersonal relations. And indeed, solidarity can only be developed when helping a persons in need is not just a routine or insensitive service, nor is it exercising supremacy over a weaker person, but it is an understanding that despite differences in strength both of the participants (carer and care receiver) are human beings equal in their human dignity.

Furthermore, if a caring relationship helps the carer to develop as a human being, growing in empathy and solidarity, the care receiver, despite his/her frailty, feels that he/she is also giving something of great value. By knowing this, the care receiver receives mental, social, and spiritual power along with the provided services for physical well-being, which enables him or her to remain a holistic human being with the defiant power of the human spirit (Frankl, 1987) despite any limitations imposed by the illness. Therefore, this way of caregiving and care-receiving acts as a two-way open relationship where both parties are giving and receiving.

To summarize, long-term care is the most significant opportunity for the development of new solidarity and quality interpersonal relations on personal, professional, and societal level in the Twenty-first century. To use this opportunity, much attention from both individual and societal levels will have to be directed toward educating people on relational human caregiving and acceptance of received care with gratitude. The method of in-group social learning, described in this article, was developed with this consideration.

## In-Group Social Learning Method and Its Use in Training for Informal Carers

In-group social learning is a method used for training informal carers. It was developed by Jože Ramovš and further refined by him and his coworkers at Anton Trstenjak Institute of Gerontology and Intergenerational Relations for quality aging and long-term care. This section begins with a brief description of the in-group social learning method since it has not been yet described in English literature and since its understanding is crucial to appreciate its importance for informal carers training. In the second part of this section, the implementation of the in-group social learning method for informal carers training will be presented, evaluated and discussed.

### Method Description

In-group social learning is a group learning method based on the narration of personal experience and personal knowledge of each participant, moderated by a group leader. The method focuses on positive experiences and includes only negative experience that were successfully resolved. It builds on the human ability to experience empathy and solidarity toward others and in turn, develops these two characteristics further. One of the most significant advantages of in-group social learning is a bidirectional link between theoretical knowledge and actual living situation—skills and knowledge needed by participants are simultaneously transferred from and to everyday practice, helping participants to further understand their needs and possible concrete solutions. Goals of the method are: development and shaping of human personality, quality intergenerational and other interpersonal relations, and encouragement of dialogue within families, working environments and on a societal level (Ramovš, 2000, 2013).

In-group social learning method was developed by Jože Ramovš using Bandura's theory of social learning, Frankl's logotherapy and Moneno's teaching about group and group work as sources. The origins of the method date back to 1980, when the author was working with adolescents and people suffering from addictions and was using it for the purpose of health and social prevention. In the following years, when his focus moved toward working with older persons, the method was extensively developed in the area of gerontology; at the beginning as part of the intergenerational programs and programs for healthy, active, and quality aging and later for the area of long-term care. As of this date, Dr. Ramovš's team at Anton Trstenjak Institute of Gerontology and Intergeneration carried out more than 300 training sessions using the method of in-group social learning.

Comparing the method with Aristotle's traditional learning methods, there are some unique aspects as well as some similarities:

*Scientific knowledge learning (episteme)* is based on rational ability, especially on the ability to memorize. Its goal is to collect objective, impersonal knowledge about things, people, events, processes, functioning, formation, and achievement. In-group social learning shares the same aspiration for the analytical insight of the goal and learning process, but unlike scientific knowledge learning, in-group social learning originates from concrete, personal experience and is focused toward it.

*Skill and crafts learning (techné)* is focused on productivity and acquisition of different competencies. Even though in-group social learning also focuses on the use of acquired knowledge for everyday life, its aim is directed more toward living this knowledge (existing) rather than acquiring something new (possessing something).

In-group social learning is therefore complementary to the methods of learning mentioned above, with the difference of being more internal (as is *practical wisdom learning (phronesis)* in Aristotle's division). Whereas, the main purpose of scientific knowledge learning and skill and craft learning is individual and social development within the field of “having everything needed for survival,” the main aim of in-group social learning is to develop a person's character and interpersonal relations, i.e., to be a person amongst people (Fromm, 1976). Or, if we paraphrase the observations of the philosopher and thinker on interpersonal relations Martin Buber, we can identify in-group social learning as a method for the strengthening of the Me-Thou (You) relations, whereas majority of other educational and teaching methods mainly lead to successful development of the Me-It relationship (Buber, 1979).

In-group social learning also could be compared to peer learning. *Peer learning* is a recent trend in seniors' programs and is used in a variety of contexts. It can be defined as the acquisition of knowledge and skill through active helping and supporting among status equals or matched companions. It involves people from similar social groupings who are not professional teachers helping each other to learn and learning themselves by doing so (Topping, 2005). In-group social learning is similar to peer learning because they both emphasize active engagement of every participant and sharing previous experiences and knowledge for the purpose of learning. Participants can be described as people coming from similar social groupings, e.g., in the case of informal carers training, the collective experience of being a carer qualifies participants as peers. However, unlike in peer learning, social group learning is led by a specially trained group leader. Moreover, while the success of peer learning depends on the quality of the contributions of the moderator and fellow participants (Clark et al., 1997), in-group social learning with its rules safeguards the red line of the meetings and protects the participants from dwelling on the negative experiences.

### Dimensions of In-Group Social Learning

#### Group and Its Power

The fundamental background of all modern group work is a family—the primary natural human group. Distinct types of contemporary groups reveal various family characteristics, such as:

- A deep emotional bond between members that gives a feeling of home or belonging and is a source of group consciousness.
- Direct communication between members.
- A relatively permanent and continuous entity that offers a feeling of security.
- Intrinsic goals, values, and norms (called the culture of a group).
- The intrinsic structure of the roles, activities, and positions of individual members.

Based on these characteristics, the group can be defined as a number of people who experience a mutual sense of belonging or share the same group consciousness, who communicate directly with each other and who have certain goals, values, and norms. Consequently, they also share a common program and have their own structure of roles and positions (Ramovš, 2000).

The pioneer of the modern group, group work, and interpersonal relations research was Jakob Levy Moreno. He discovered that a group presents itself as an indispensable factor in the formation of human personality and interpersonal relations and as such can be useful as a therapeutic tool (for this purpose he developed group psychodrama) and a fertile field for human relations research (as reflected in his development of the psychometric method). Moreno was an expert in the exploration of the fine balance between an individual human being, a group, and a community—the concept which the in-group social learning tries to incorporate in its practice.

A classical researcher of the group was Kurt Lewin, who studied the effect of several types of leadership on group dynamics between 1937 and 1949. This was the time when self-organized self-help groups started to form (such as AA). His research enabled a better understanding of group dynamics and group processes, its development, and different methodologies for its use. Professional and self-organized group work slowly became popular at the end of the last century, and multiple theories of group work have been established, with all of them stressing the importance of group methodology for more intense forms of learning.

As indicated earlier, group learning is social learning. For people, entering distinct groups (from family to working groups and groups of friends) also presents a learning process. Social learning was widely explored by Albert Bandura (1977). He emphasized its instrumental nature and found it to be effective through cognition driven process of experience transformations where people learn not only from their own experience but also by observing others. Researchers of social learning up to date have mainly focused on the investigation of social processes and short-term goals. Development of social learning for the field of quality aging, long-term care, and intergenerational solidarity is in many ways different. It demands an understanding of the complexity of the life processes and the achievement of long-term goals.

In our practice, the in-group social learning is usually carried out in mid-sized groups (9 to 18 participants), and rarely in small groups (2 to 8 participants) or big groups (19 to 32 participants). With such numbers, the group can be used as a safe environment where people can share their experiences and learn more intensely (from each other, from pre-prepared materials or from the experts visiting the group); it also presents a bridge between a person and a community, as described above.

#### Concrete Needs, Abilities, and Experiences of the Group Participants

The main motivation for participation in the in-group social learning is concrete, personal need of the participant. At the same time, a participant's abilities and experiences present the best starting point for working with the group. Building on this, the topic of the program is always focused on something concrete; e.g., training for family and other informal carers revolves around topics such as mastering the basic nursing skills, skills for communication with disabled older persons, coping skills, and empowerment for a healthier lifestyle. Furthermore, training manuals are structured in a way that incorporates modern knowledge on the addressed subject and helps participants to integrate acquired knowledge with their own experience. To enable this, much of the time during the meetings (three quarters) is dedicated to experience sharing; if possible, the participant should have an opportunity to voice his/her experience or thoughts two to three times per meeting.

Experiences are a crucial factor for the in-group social learning. Experiences include everything an individual did in his/her life, everything he/she experienced and everything that has happened to him/her. They are the primary determinant of our current experience of the world, the actions we take, and our orientation for the future. Moreover, that goes for all experiences: bad and good, common and exceptional, conscious, and unconscious. Experiences are the most private property of a human being, as personal as his physical body. They make us who we are: happy or unhappy, good or bad, agreeable or unpleasant to others, successful or unsuccessful. But not solely so in their original form, but mostly when they are processed by our brains through communication and in relation with others (Ramovš et al., 1990; Laing, 2015). In the fast tempo of modern life, there is a tendency for good experiences to sink into forgetfulness, while bad experiences remain inside our consciousness and become sharper as time goes by. In order for bad experiences to find a useful place in human life, they have to be properly processed.

In-group social learning facilitates the development of human experience. Group with specially trained group leader helps the participant's cognitive process to stimulate good experiences stored in the memory. Moreover, healthy recognition of positive experiences is a requirement for successful learning and processing of the negative experience into important findings and effective new behaviors (Ramovš, 2013). For this demanding process, adapted methods from modern positive psychology and psychotherapy are used in the in-group social learning.

For personal growth and responsible development of human relations, experiences of other people are almost of equal importance as our own experience. When one listens to another person, when one observes him or her with openness, affection, and empathy, one learns from his or her experience almost as much as from their own experience; a child, for example, learns mainly through imitation (Bandura, 1962). At the same time, our experiences are most successfully integrated into our development when we share them with others inside a safe social environment that accepts us openly and attentively (Manski, 1993; Pellerey and Grzadziel, 2011). The in-group social learning can be such an environment, both for sharing experiences and for openly listening to the experiences of others. In our experience, deciding factors that enable this are: topic that is perceived important and necessary to all participants, as described above, and effective communication culture, which will be explained in greater detail later.

#### The Role of Each Participant

In the group social learning, personal development of the participant and his/her acquisition of practical skills depends on his/her active role inside the group. Roles of the participants within the group are based on their actual needs in real life and not on the group learning program. They cater to the individual ability of each participant. During the group activity, the leading role is given to the person sharing experience or expertise. This role must be given to each group participant at least one time per meeting, enabling the participants to share something they perceive as important, resulting in their own satisfaction, which is reflected in the satisfaction of other participants, who strive to listen with interest and affection.

The hidden risk of the in-group social learning is collective ideologization. Resulting from group program and/or a bad group leadership and atmosphere, it can pull all the participants into a collective process that does not answer participant's individual, concrete needs and does not help with his/her development (Mastromarino, 2013). If this occurs in our case, the informal carers cannot learn to provide better care for their care receivers nor can they develop personally; the only thing that is possible for them in such an environment, is developing into some sort of a “specialists,” as defined by the ideology imposed by the program, or into a replica of the group leader.

A second latent risk for the in-group social learning is individualistic spontaneity of the learning process. It occurs if group dynamics are left in the hands of a coincidental individual who (mostly subconsciously) uses the group for the gratification of his/her own needs and exhibition of his/her own abilities, without addressing the goals and needs of the participants (Mastromarino, 2013). Consequently, group participants become increasingly dissatisfied with the program and their role in the group, the atmosphere deteriorates, and an increased drop-out rate of people with an expectation to learn and equally participate in the training is observed.

These potential pitfalls in the in-group social learning can be avoided by:

- A clearly prepared program accessible to all participants and introduced by the group leader (e.g., during the informal carers training specially prepared manuals are used for this purpose; they are given to each participant at the beginning of the training and followed chapter by chapter during the training);
- Rules of effective communication culture within the group, which are clearly stated and known to all participants;
- Competent and responsible moderation of the group by the group leader, in a way that integrates the criteria mentioned above.

In-group social learning methodology is, therefore, neither authoritative nor anarchic, but democratic. It predicts communication between all participants in an orderly manner, with well-defined roles, and with an understanding that each participant bears responsibility for the quality of his/her role.

Working with a methodology of in-group social learning, group leadership demands special knowledge about group work, holistic understanding of a person, and the ability to encourage positive experience in social learning. To achieve that, a group leader has to undergo a special training consisting of theoretical and practical knowledge. As part of the training, he/she also learns how to bring part of his/her story and experience into the program. All of the group leaders up to date have been trained by the author of the method or by one of his coworkers.

#### Effective Communication Culture

The most crucial tool in reaching group goals during social learning is communication. The quality of communication culture will determine the quality of learning and the atmosphere of the group. In line with the axiom “one cannot *not* communicate,” defined by the classic communication researcher Paul Watzlawick, the quality of learning and the atmosphere of the group will be also influenced by nonverbal communication and the acts of participants in the group aimed at each other and/or at the group as a whole (Watzlawick et al., 1967).

Because investment in effective communication culture within the group helps to achieve other goals of social learning, four standard rules for effective communication culture are provided at the front page of each training manual and repeated at the beginning of every training (and in between if needed). The rules are:

1. We attentively listen to everyone without interrupting them.
2. We talk about things within the topic that the group is currently discussing.
3. We share personal experience in the first person singular.
4. Whatever personal information people share within the group, should stay within the group.

During group orientation, these rules are further concretized through practical advice on excellent quality sharing and listening, advice on personal approach toward speakers, and on meaningful silence. The rules for effective communication we use, are based on research observations steaming from the couple's therapist (Engl and Thurmaier, 1995), positive psychology observation, according to which there should always be several more positive messages than negative ones (Gottman and Declaire, 2001) and active research observations from our practice of in-group social learning.

In-group social learning uses three types of communication: 1. light and pleasant chatting; 2. functional, informative talk; and 3. sharing of personal experiences, realizations, opinions, and emotions. First two are needed in the group for organization and development of atmosphere, while the third one—sharing and listening to the experiences and other deeply personal topics—plays a role in personal development and development of interpersonal relations of the participants.

Effective communication culture in the group exhibits the following traits:

1. Each of the participants can tell and show what they think, feel, or consider meaningful in a clear and polite way.
2. Each participant exhibits empathy and puts effort into communicating his/her thoughts in a respectful way, and in a manner that is understood as they would wish to be themselves.
3. Each of the participants attentively listens to others, so that he/she comprehends what the person speaking wanted to communicate, and therefore not what he/she might assume that the speaker wanted to communicate.
4. All the participants are willing to discuss common things in a way that eventually results in a consensus.

#### The Enthusiastic Atmosphere in the Group Stimulated by Encouraging Topics

The topic of enthusiasm has become one of the main focuses of modern neuroscientists specialized in pedagogy. Gerald Hüther calls it “doping for mind and brain” (Hüther, 2016). The reward system, a collection of brain structures and neural pathways that are responsible for reward-related cognition, are crucial for the feelings of happiness, energy and motivation, needed for working and learning. A pleasant experience is likely to be repeated, resulting in a formation of new neuronal connections in the brain, enabling the preservation of newly acquired skill or knowledge. Such neuroscientific findings can explain the motivational power of good experiences and the enthusiasm about the positive actions of others for the successful learning (Rizzolatti and Craighero, 2005). They are in line with an ancient observation that enthusiasm or happiness makes people more perceptive emotionally and cognitively.

For a better understanding of the in-group social learning, it is important also to note the findings of the mirror neurons and the role they have in empathic behavior toward others (Iacoboni, 2009). These neuronal cells facilitate empathy toward the person that is responsible for evoking the feelings of enthusiasm. While observing such a person, our brain reacts similarly as if we were enthusiastically performing the act ourselves; in both cases (when mirroring the enthusiasm of others or when experiencing it personally), the learning process feels easy and is successful. Therefore, the relationships that make us feel enthusiastic, result in spontaneous imitation of other person's behavior and internalization of his/her experiences. Such learning ability is especially prominent with young children. However, with the conscious training focused on meaningful experiencing and communication within a group, we can retain and further develop it also later in life.

Abilities to sympathize, empathize, and identify with another are crucial for the development of high-quality interpersonal relations and solidarity (Ryff, 1995). Even the abundance of rationally accumulated knowledge about human relationships or sincere declarations about the importance of acceptance and equality is ineffective if there is an absence of empathy in interpersonal and collaborative relations. Personal development and strengthening of interpersonal relations are predominantly rooted in enthusiasm and joy about other people, about their concrete actions, characteristics, experiences and plans. As mentioned before, this leads to the development of the brain into an efficient tool for internalization of experiences and knowledge. The brain that remains dynamic, continuously shaping and upgrading, makes such development possible also in later age. In line with this, neuroscientist (Spitzer, 2012) compares the brain to the building site, where the things we experience build and strengthen new neuronal connections and networks.

The in-group social learning method helps people retain some of the curiosity, openness, and enthusiasm of childhood. In the circumstances imposed by today's civilization, a person's ability to be enthusiastic about things and people often dulls over time and dies off or takes a turn for pathological development (Freudenberg and Samarkovski, 2014). Both of these traits are fatal for mental, social, spiritual, and also healthy physical personal development and good interpersonal relations. Motivation and enthusiasm are needed throughout all developmental stages of our life and are no less important during our middle and late years as they are during our childhood. Through the in-group social learning people learn to consciously direct attention toward the kind and positive things about themselves and about others. This is important since the focus on the positive facilitates the finding of concrete solutions to the urgent needs of the participants and, in combination with effective communication culture, creates a pleasant and encouraging atmosphere, the importance of which was described above.

The in-group social learning takes place whenever discoveries and experiences are flowing freely among all of the participants in a group. During the development of this method, the team attached a motto to this dynamic process: “all of us are teachers and all of us are students; our differences enrich us.” Furthermore, the realization that acceptance and giving are inextricably linked with one another is expressed in the second motto: “If you want to look after Yourself, help the Other; if you want to look after the Other, take care of Yourself!” In such a learning environment, informal carers can learn skills, come to realizations, and shape opinions that enable them to take easier and better care while effectively not forgetting themselves.

### Implementation of the In-Group Social Learning Method for Informal Carers Training

The current training program and practical aspects of the in-group social learning methodology are described in a manual used both by leaders and participants—informal carers—during the training (Ramovš and Ramovš, 2018a), and has been presented in Slovenian academic and other publications (Ramovš and Ramovš, 2018b). To preserve its quality, the model is protected by copyright.

Usually, there are around 20 participants. Each of the ten sessions lasts for 2.5 h. And each session is specially dedicated to one of the challenging themes of home care.

The themes are divisible into four general areas:

1. **Understanding of an older family member and communication with him/her**. More than one-fourth of the whole program is dedicated to the skills for better understanding of disabled person and communication with him/her, which is particularly important when the behavior is troublesome or socially difficult due to the illness (e.g., in dementia) (Eggenberger et al., 2012). Humane relationships and communication are the weakest points in the development of modern European long-term care. Understanding that communication is entirely the consequence of a person's perception is a basis for our training and care practice. By directing our attention to a positive aspect of another person, positively experiencing him/her, we exercise our human freedom to the fullest. Various techniques for positive interpersonal perception have been developed as part of the training and participants are encouraged to use them not only during the training but also daily while providing care. The evaluation results show that these topics were the most useful for the participants.
2. **Nursing skills and conscious care for oneself**. Professional physical therapists and nurses take part in the training sessions. The participants learn about practical nursing skills helping them to perform ADL and IADL for the care receiver. These tasks can be physically and mentally very demanding for carers, and the latest professional findings can make them significantly easier. Information about various aids and materials (e.g., nursing bed or adult diapers) and where to purchase them are equally important. Part of the training is dedicated to the preservation of carers' health. Strengthening their physical condition, learning stress and anger management as well as maintaining a sensible attitude in difficulties situations and thinking about timely inclusion of other family members are for carers very important health prevention habits that can be considered and nurtured.
3. **Age-related diseases, dying and grieving**. In each group, there are carers dealing with dementia of their family member. Therefore, one of the sessions is dedicated to the understanding of this illness and to the necessary communication with such an individual (using validation technique). Heart attack and brain stroke can also be reasons for care, as well as hip fracture and another chronic physical, mental, and social illnesses related to aging. In each group some family members have their relatives already in the terminal phase of the illness while others are expecting or even fearing the progress of the illness of the person they care for, making it necessary to talk also about palliative care, dying and grieving.
4. **Resources for carers and formation of local family carers' group**. Resources for carers are rapidly developing. There are day, night, and short-term care services now available. These programs are respite support for informal carers, and with their development, synergistic complementarity of formal and informal care is forming, for the benefit of all. Realistic options and possibilities for the respite of carers within local communities are explored as part of the training. The exchange of experiences on this subject give carers strong psychosocial support and motivation. The option of transfer of the care receiver from home to institutional care is also reflected on in training since it is usually a difficult decision for nearly everyone.

The last session is dedicated to the establishment of a local family carers' group, based on the concepts of self-help and self-organization. Two members of every group are carefully chosen and trained to become group leaders. The family carers' group has sessions once per month, offering to family and other informal carers support in caring and grieving. The supervision work is provided by the Institute, connecting informal carers into a national network of informal carers. The goals of the local groups for family carers are:

1. Mutual support during the often-demanding caregiving period.
2. Continuous training for the provision of quality care in the light of the realization that care receiver's life is coming to an end and that good relationships and communication are central to good care.
3. Support during the grieving period.

When caring and grieving periods are over, many carers become volunteers for good quality aging and intergenerational coexistence in their local environment.

The main prerequisite for successful group leadership of the training for family and other informal carers is proficiency in the in-group social learning method. Limiting the training to lectures and discussions does not justify the time for the participants and costs of the training, as this knowledge is mostly freely available on the internet. Therefore, it is vitally important to actively involve all participants to share their respective experiences and to provide for a good atmosphere that offers emotional relaxation and support as well as strengthens motivation. The participant with an unsolved problem always has a priority in the group process. In the method of in-group social learning, the concept of problem-solving is concrete, personal, and sharing oriented. The latter is achieved mainly by asking: “*Is there anybody experienced in solving a similar problem?”*

It is of critical importance that professional presenters impart their knowledge in a manner that is respectful of the carers' context and learning interests. In cases where presentations dwell on theory and do not engage with practice and carers' experiences, the carers express futile criticism and negative attitudes. The starting point and the goal of the in-group social learning is effective two-way dialogue between any professional knowledge and any experiences of participants. This can be achieved by good cooperation of group leaders and experts in chosen themes. A group leader trained in the in-group social learning methodology is always assigned for the course of the training. The leader proceeds by inviting experts for specific themes. Each session starts with the so-called “in-circle sharing” where all participants share their respective experiences (if any) concerning the chosen theme. The process is facilitated by the group leader, while the expert listens carefully. This is followed by an interactive presentation of the theme by the expert or by his/her demonstration of required skills. The session ends with a discussion between participants and expert, allowing additional questions and worries to be addressed.

Up till now, close to a hundred family and other informal carers training courses were carried out across Slovenia in Croatia. Numbers of participants attending have risen yearly. After finishing the first course on a local level, positive feedback often impels local authorities to ask for another round.

The next section will explore other benefits and some shortcomings of the in-group social learning method for training informal carers.

## Evaluation of the In-Group Social Learning Methodology in Informal Carers Training

In this section, the evaluation study is presented and discussed. To evaluate the in-group social learning method used for informal carers training, a combination of objective and subjective data analysis was performed. Training participants gave a quantitative evaluation of the training in general, of being able to express themselves during the training and of being acknowledged by other training participants—segments very important for the effect of the in-group social learning, as described in the previous section. Furthermore, participants provided a qualitative evaluation of their personal training benefits. As the nature of informal caregiving is very personal and since the in-group social learning method aims at the development and shaping of human personality in a safe, intimate environment, subjective data about personal benefits are of great relevance to gain insight into the efficacy of the training.

For some aspects, evaluation study data were compared with the corresponding data from the national representative research: Aging in Slovenia—Survey on the needs, abilities, and standpoints of the Slovene population aged 50 years and over (Ramovš et al., 2013). This was done for the following areas: analysis of demographic, health, personal experience with care receiving, opinion on informal carers training necessity, and desired way of care receiving. These areas were chosen since their comparison with evaluation study data provides further insight into benefits and shortcomings of the in-group social learning method used for informal carers training as well as indicates potential guidelines for its further development.

### Method

#### Participants

The sample consists of the informal carers that took part in one of the 28 “Trainings for Family and Other Informal Carers financed by Ministry of Labor, Family, Social Affairs and Equal Opportunities” which took place between the years 2010 and 2018 in various towns and boroughs of Slovenia. The sum totaled in 453 individuals (age 23–83; *M* = 56.00; *SD* = 10.77; 92% female).

National representative research of aging in Slovenia originally included 1047 participants (age 50–98; *M* = 66.08; *SD* = 10.59; 59% female) who were determined by Statistical Office of the Republic of Slovenia. As our aim was to compare the data of informal carers that underwent the in-group social learning method used for informal carers training, to non-trained informal carers, all the informal carers among the participants of representative study were selected, resulting in a sample of 200 individuals (age 50–98; *M* = 67.37; *SD* = 10.95; 55.4% female) (Ramovš et al., 2013).

#### Study Procedure

For data collection, a questionnaire in printed form was used. Participants completed it at the end of the training they participated in. The questionnaire was made up of two sections of items: the first section has been initially part of the instrument, used in aforementioned national representative research of aging in Slovenia, while the second was developed purposely for the training's participants in order to measure their personal experience obtained during the training. The questionnaire with both sections was evaluated by Social Protection Institute of the Republic of Slovenia that also evaluates programs financed by the Ministry of Labor, Family, Social Affairs and Equal Opportunities in the Republic of Slovenia.

In the first section of the questionnaire, the items collected information about informal carers—their demographic data, health status, personal experience with care receiving, opinion on informal carers training necessity and desired way of care receiving.

In the second section of the questionnaire, four training evaluation items were used. One item asked the participant to describe with their own words, which content of the training they consider most useful for themselves. Other items were used to assess the participant's general evaluation of the training, to what degree they felt they could express themselves during training, and to what degree they felt they were acknowledged during training.

#### Data Analysis

Paper and pencil survey approach was used for data collection. Quantitative data analysis (Chi-square estimations, *t*-test, and descriptive statistics) was done using Excel software. For qualitative data analysis thematic analysis was used following the steps identified by Braun and Clarke (Braun and Clarke, 2006): data familiarization through reading and re-reading; systematically generating initial codes across the whole data set; identifying themes within identified codes; reviewing themes for internal and external validity; and defining and naming themes. One member of the research team (MR) who had not been involved in the development of the questionnaire, conducted the initial analysis. After the initial analysis was complete, the codes and themes were reviewed and revised by a second and third researcher (JR and AS). The revised analysis was then discussed until consensus was reached.

#### Informed Consent

All subjects within this sample of participants were informed about the aim of the study and voluntarily answered the items of the questionnaire if they decided to participate in the study. Written informed consent, which also applies to the use of the data for research purposes, was gained from all the participants before data collection. Participation was anonymous.

## Results

### Gender

In the training participants sample of informal carers the female domination is evident (Table 1), while in the Slovenian representative sample, the proportion of female and male informal carers are more equally distributed. The difference is significant [X^2^(1, *N* = 646) = 141.13, *p* < 0.01)].

**Table 1:** Training participants sample of informal carers by gender and its comparison to the representative sample.

**Informal carers:**		**Male**	**Female**	**Total**
Training participants	Frequency	26	418	444
	Percentage	**5.9%**	**94.1%**	**100.0%**
Representative sample	Frequency	91	111	200
	Percentage	**44.6%**	**55.4%**	**100.0%**

### Age

**Figure 1:**
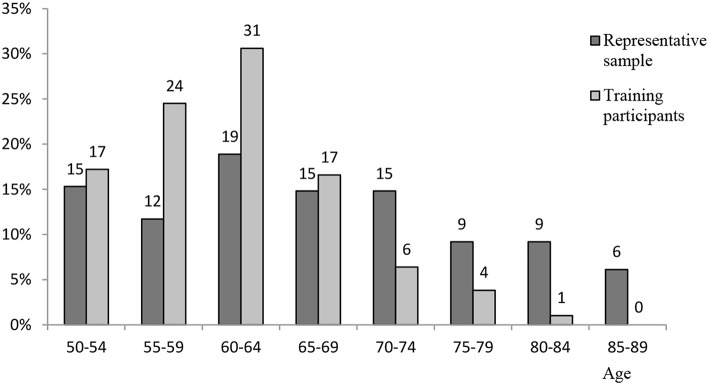
Training participants sample of informal carers by age and its comparison to the representative sample.

The average age of the training participants informal carers (*M* = 56.0, *SD* = 10.8) is much lower than the Slovenian representative sample of informal carers (*M* = 67.4, *SD* = 11.0). As the *t*-test has shown, the difference is significant [*t*_(631)_ = 14.04, *p* = 0.000]. Given the fact that this difference is affected by the differences in the sampling, as the representative sample participants were aged 50+, whereas 30.7% of the training participants were younger than 50, the age-group comparison is more relevant. The age-group comparison of both samples' participants is given in Figure 1.

Percentage of informal carers in age-groups are more equally distributed in a representative sample, while in the training participants sample a higher percentage of younger than 70, and a lower percentage of older than 70 can be observed.

### Employment Status

The comparison provided in Table 2 shows the employment status differences between two samples of informal carers. The differentiation was made in two categories—a full-time job (employed) and not employed (unemployed, retired, occasional, or part-time job). This type of differentiation is appropriate for the distinction between informal carers, who are mostly experiencing an intensive lack of time for quality caregiving and informal carers, who usually have enough time for caregiving, but often experience lack of financial income. The results show significant differences between both samples—among training participants informal carers there is a much higher percentage of full time working carers [X^2^(1, *N* = 594) = 9.30, *p* < 0.01].

**Table 2:** Training participants sample of informal carers by employment status and its comparison to the representative sample.

**Informal carers**		**Full-time job**	**Mostly at home**	**Total**
Training participants	Frequency	123	279	402
	Percentage	**30.6%**	**69.4%**	**100.0%**
Representative sample	Frequency	36	156	192
	Percentage	**18.8%**	**81.2%**	**100.0%**

### Civil Status

In Table 3, the percentage of single carers and carers with partners are shown in both samples. The difference is small and is not significant [X^2^(1, *N* = 638) = 0.34, *p* > 0.05].

**Table 3:** Training participants sample of informal carers by civil status and its comparison to the representative sample.

**Informal carers:**		**With partners**	**Single**	**Total**
Training participants	Frequency	325	113	438
	Percentage	**74.2%**	**25.8%**	**100.0%**
Representative sample	Frequency	144	56	200
	Percentage	**72.0%**	**28.0%**	**100.0%**

### Education

Informal carers that participated in the training have significantly higher education compared to the carers in a national representative sample, that did not participate in training for quality informal care and this difference is important [X^2^(4, *N* = 639) = 49.75, *p* < 0.01]. The percentages are shown in Table 4.

**Table 4:** Training participants sample of informal carers by education level and its comparison to the representative sample.

**Informal carers:**		**Primary**	**Secondary**	**Tertiary**	**Total**
Training participants	Frequency	47	245	147	439
	Percentage	**10.7%**	**55.8%**	**33.5%**	**100.0%**
Representative sample	Frequency	65	99	36	200
	Percentage	**32.5%**	**49.5%**	**18.0%**	**100.0%**

### Health Status

The differences in health status among a representative sample of carers and training participants are not significant [X^2^(4, *N* = 633) = 4.46, *p* > 0.05]. As shown in Table 5, more than half of the carers from each sample experience some health problems.

**Table 5:** Training participants sample of informal carers by health status and its comparison to the representative sample.

**Informal carers:**		**No problems**	**Moderate problems**	**Major problems**	**Total**
Training participants	Frequency	166	256	16	438
	Percentage	**37.9%**	**58.4%**	**3.7%**	**100.0%**
Representative sample	Frequency	91	99	5	195
	Percentage	**46.7%**	**50.7%**	**2.6%**	**100.0%**

#### Personal Care Receiving Experience

Among the training participants sample, there are significantly more carers that have experienced being a care receiver themselves compared to Slovenian representative sample of informal carers [X^2^(4, *N* = 626) = 10.7, *p* < 0.01]. The results are presented in Table 6.

**Table 6:** Training participants sample of informal carers by personal care receiving experience and its comparison to the representative sample.

**Informal carers:**		**None care receiving experience**	**Care receiving experience**	**Total**
Training participants	Frequency	336	91	427
	Percentage	**78.7%**	**21.3%**	**100.0%**
Representative sample	Frequency	178	21	199
	Percentage	**89.4%**	**10.6%**	**100.0%**

#### Desired Way of Care Receiving

Compared to the Slovenian representative sample, more carers in the training participants sample would choose informal care rather than institutionalized when given a choice [X^2^(1, *N* = 575) = 4.66, *p* < 0.05]. The results for the desired way of care receiving can be found in Table 7.

**Table 7:** Training participants sample of informal carers by desired way of care receiving and its comparison to the representative sample.

**Informal carers:**		**Informal care receiving**	**Formal care receiving**	**Total**
Training participants	Frequency	262	124	386
	Percentage	**67.9%**	**32.1%**	**100.0%**
Representative sample	Frequency	111	78	189
	Percentage	**58.7%**	**41.3%**	**100.0%**

#### Opinion on the Necessity of Training Participation for All Informal Carers

Both sample participants also answered the item, asking them about their opinion on the necessity of training participation for all informal carers. As seen from Table 8, a higher number of training participants expressed there was a need for training compared to the informal carers in Slovenian representative sample; the difference is significant [X^2^(1, *N* = 630) = 25.22, *p* < 0.01].

**Table 8:** Training participants sample of informal carers by opinion on the necessity of training for all informal carers and its comparison to the representative sample.

**Informal carers**		**Training necessary**	**Training not necessary**	**Total**
Training participants	Frequency	437	2	439
	Percentage	**99.5%**	**0.5%**	**100.0%**
Representative sample	Frequency	175	15	190
	Percentage	**92.1%**	**7.9%**	**100.0%**

#### Informal Carers Relation to Care Receiver

**Figure 2:**
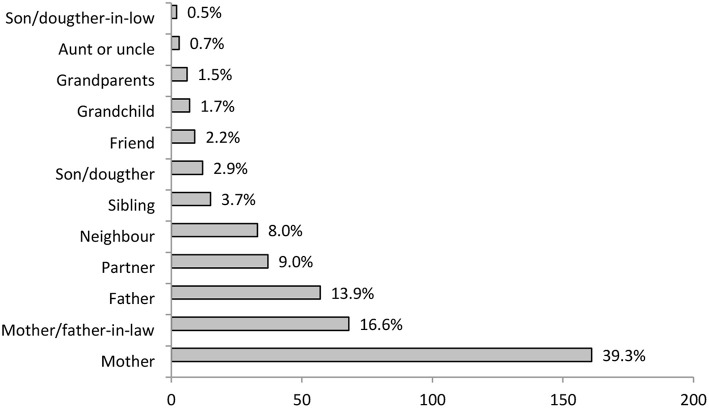
Training participant informal carers relation to care receiver (*N* = 410).

The first item in the second section included information about who are the care receivers of care provided by the training participants. As it is evident in Figure 2, most of the participants are caring for their mother and many for their father or parents-in-law. Some of them are caring for their partner or their neighbor; other answers were far less common.

#### General Evaluation of the Training

**Figure 3:**
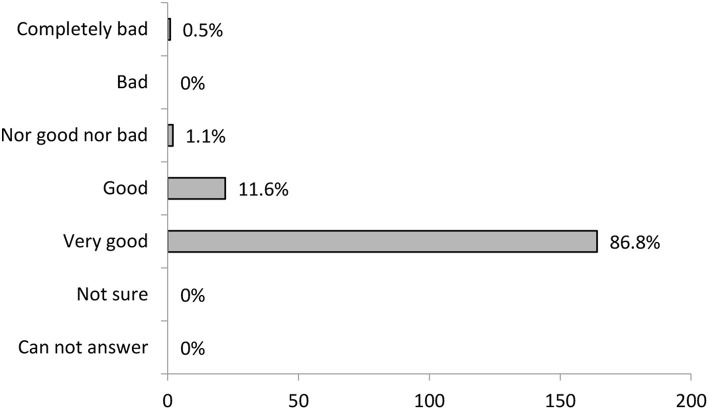
Training participant's general evaluation of the training (*N* = 189).

The general evaluation of the in-group social learning method-based training by informal carers was very positive (Figure 3). The participants mostly responded with the subjective general evaluation answer “very good,” the majority of others viewed the training as excellent. Other possible answers were chosen by 1% of the participants or less.

#### Expressing Themselves on the Training

**Figure 4:**
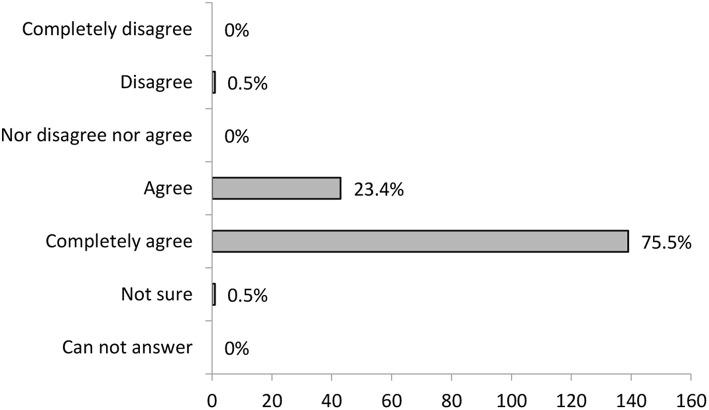
Training participant's evaluation on how much they felt they could express themselves during training (*N* = 184).

Next evaluation result is summing the training participants' feedback on how much they felt they could express their experiences and suggestions during the training (Figure 4), which is a very important part of the in-group social learning methodology. The majority agreed that they could express themselves. The negative evaluation was rare.

#### Being Acknowledged During the Training

**Figure 5:**
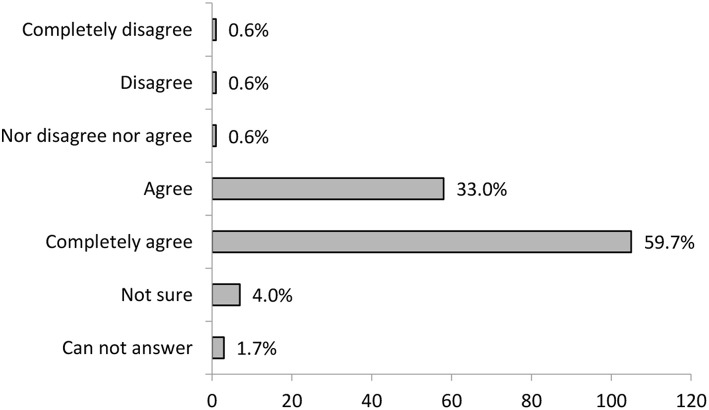
Training participants evaluation on how much they felt they were acknowledged during training (*N* = 176).

The participants on informal carers training were also asked to evaluate how much they felt other participants acknowledged their expressed experiences and suggestions during training. The importance of this was presented in the in-group social learning methodology section. The results are shown in Figure 5. As is evident in the diagram, the majority of participants agree or agree entirely that they felt acknowledged during the training.

#### Perceived Personal Benefits of the Training Participation

In Table 9, the categories of perceived personal benefits are presented. As described in the data analysis section, a whole data set was coded and divided into four categories. Many of the participants stated more than one category in their answers. The first three categories are the most relevant for the evaluation of the in-group social learning method, and their contribution will be further presented in the discussion.

**Table 9:** Training participants perceived personal benefits from training.

**Category**	**Prevalent content**		**Frequencies**
1	Knowledge and skills		296 (37.7%)
		Positive communication	
		Nursing	
		Understanding diseases and dementia	
		Palliative care	
		Passing away and grieving	
		Understanding old age	
2	Experiences exchange		179 (22.5%)
		Sharing own experiences	
		Comprehending experiences of others	
		Relating to others' experiences	
		Collective group experiences	
3	Inner strength		168 (21.2%)
		Self-confidence	
		Self-confirmation	
		Social support	
		Social inclusion	
		Stress relief	
		Health strengthening	
4	Approval of training		151 (19.0%)
		Contentment with the training	
		Praise of training's quality	
		Praise of training's guidance	
		Gratitude for training participation	
		Appreciation of themes	
		Total	794 (100.0%)

## Discussion

Several items (1–6 and 10) of the evaluation study collected demographic data, data on participants' health status and their relation to care receiver. Even though these items do not have direct importance for the evaluation of the in-group social learning methodology used for informal carers training, they provided information about the group that has been evaluated. On account of national representative research that collected the same items in almost the same period in a separate sample, it was possible to compare training participants to Slovenian representative sample of informal carers. Since described items were part of the evaluation study, and their results influenced the informal carers training and the development of the methodology, they will be shortly discussed here.

Looking at gender distribution, the results showed a considerable difference in the presence of males between training participants and representative sample. A relatively high percentage of male carers in Slovenian representative sample of informal carers in comparison with other studies (Arber and Ginn, 1995; Dahlberg et al., 2007) and data (Central Statistics Office Ireland, 2012) could be an outcome of the fact that men, unlike women, are more aware of their caregiving. In Slovenia, the patriarchal social patterns are still very present, and women caring for the family members in need is often self-evident. On the other hand, due to these patterns, men might also find it harder to admit that they need help with providing care, which could explain their significantly lower participation rate in training. This area should be further explored with the aim to better understand the role of female carers; however, given the fact that there is a substantial amount of male carers, it is evident that we should aim to find a way to include them in training for informal carers.

According to presented data, there are many carers in Slovenia older than 70 or even 80 years, who mostly do not participate in the training. It is possible that training participation is harder for them due to the higher prevalence of health-related issues among these generations (Ramovš et al., 2013; SURS – Statistical Office of the Republic of Slovenia, 2019). This shows the importance of earlier preparation for caregiving, when one is still capable of participating in the training; as described at the beginning of the article, even if there are no caring responsibilities yet, the likelihood for a person to become carer later in life is getting more significant with population aging, so training should be considered in earlier periods.

Younger carers usually do not have as many health-related problems as older carers. However, they often lack time and experience increased stress levels due to caring responsibilities toward their growing children in combination with full-time employment (Riley and Bowen, 2005). For that reason, employment status comparison was added in this section. Interestingly, the data showed that full time employed carers more often participate in the training compared to unemployed carers. This trend can be explained by the younger age of employed training participants; besides that, employed carers are likely to be better socially included, more informed about the possibilities in their community as well as are likely to be better organized, which helps them to better manage their time. The area should be further explored. However, attention was already given to the number of training sessions needed and time of the day when trainings are conducted, making it possible for the employed carers to participate as well. Despite the ratio difference, the percentage of full-time employment among both samples of carers is still very low, which is consistent with other research results. Informal carers often find themselves in a situation, where they are forced to leave their jobs to be able to care for their family member properly. Even though those carers have more time, they are potentially experiencing a lack of financial income (Pitsenberger, 2006). This knowledge together with the realization that employment status of the training participants differs (which consequently leads to different experiences of caregiving), is very important for the informal carers training group leader, especially when he or she uses the in-group social learning method to address the topics of work-life and care-life balance. This knowledge was also used in the preparation of the training manual.

Furthermore, the training participants usually have a higher level of education, which could be connected with the notion that higher education is often connected with greater awareness of the importance of educating, training, schooling, learning, and obtaining knowledge and skills. Furthermore, higher educated persons may be better at finding information about the training. The area should be further explored; however, given the fact that there is a substantial amount of less-educated carers, we should aim to find a way to include them in training for informal carers.

Assessing the health of informal carers, there were no relevant differences between the two samples. Nevertheless, the results are providing insight into general Slovenian informal carers' health status: more than half of them are experiencing their own health issues. This indicates the potential adverse effect of caregiving on carers health. Some authors claim that the more the caregiving responsibilities are demanding, the more negative effect they potentially have on the health of a carer (Schulz and Beach, 1999), while others argue the complexity of the situation (Beach et al., 2000). However, combining all with just mentioned research data, informal carers training program incorporates topics of empowerment for a healthier lifestyle and care for oneself and should aim to do so in the future.

Data about the identity of the care receiver presented in Figure 2 is comparable to Slovenian representative sample data. Similar to most of the European countries (European Commission, 2012), the majority—close to 90%—of care receivers are family members. In line with this and built on practical experience that cares can better self-identify that way, Institute prioritizes the term “family carers” over “informal cares” when working with carers. At the same time, special attention is given to the inclusion of non-family carers (e.g., by naming the training: Training for family and other informal cares). However, for this article, authors consciously decided to use a term informal cares, since it is more comprehensive and increasingly recognized by European institutions.

In contrast with the discussed items, providing valuable information about the carers, the remaining items provided information about their opinions, experiences, and their evaluation of informal carers training. Items seven and nine provided information about the perceived need for informal carers training. Although the number of informal carers that perceived training as strongly needed was high in both samples, it was significantly higher in the sample of training participants. Taking into consideration that the latter have actively decided to participate in the training and mostly had very good experience with it (as can be seen from the results of the evaluation study), such difference is not surprising. More important is a finding that comparing the data, informal carers participating in the training had in significantly higher percent previous personal experience of being a care receiver. It seems likely that carers who know how it is to be a care receiver more often decide for training participation because they are more aware of the importance of quality care and are therefore willing to invest more effort into achieving it. The subject should be further explored though since it presents possible leverage for training motivation both for carers and authorities.

The last four questions focused directly on the above-mentioned evaluation of the in-group social learning methodology used for informal carers training. General evaluation of the training was very positive. This was shown by the participants' answers on general evaluation item as well as by the qualitative analysis of the answers on personal benefits of the training. In the qualitative analysis, a positive attitude toward a holistic experience of the training appeared as one of the four relevant categories.

Another point of view on the efficiency of the training was provided by the participants' feedback about how much they felt they could share their experiences with others during the training and how much they felt their experiences were acknowledged. The most frequent response was that they agree they felt they could share their experiences and that they agree that what they shared was acknowledged. As explained in detail in the previous section, both activities are essential for the good outcome of the in-group social learning. Their importance was also recognized by training participants, which could be seen from the fact that experience exchange appeared as one of the four relevant categories in the qualitative analysis of the last item. In particular, the experience of being acknowledged for their work gives the informal carers an important recognition in their often-new role and strengthens their motivation for the future. This was also reflected in the inner strength section of qualitative data analysis, where participants highlighted self-confirmation and self-confidence as perceived benefits of the training.

As previously indicated, qualitative data analysis provided the biggest insight into the efficiency of the in-group social learning methodology for this type of training. Participants' perceived personal benefits were divided into four categories. Three of these categories were in close relation to the expected benefits of the in-group social learning method—knowledge and skills, experiences exchange, and personal development in the form of inner-strength.

While it could be argued that we did not objectively measure the amount of acquired knowledge and an increase in communication and other skills, it is clear that participants in great number recognized the importance of positive communication, of understanding the old age and of possibility to acquire knowledge on diseases and palliative care. Since the recognition of knowledge importance is a prerequisite for any successful learning and skill acquisition, we believe it is safe to assume that they have successfully learned something. The likelihood of that is even bigger if we take into account the enthusiastic predisposition toward knowledge of the majority of the participants, importance of which for the learning process was described in the previous section and that could be observed from following examples of answers.

“*I liked exchanging the experiences because the experiences are real, and you can learn from them better.” participant 344*.

“*I liked the training because it was based on personal experiences and narration of those. From experiences you can learn the most, theory often fails you in practice, in concrete situations” participant 153*.

These two and other representative answers also showed how the in-group social learning helped people bridge the theory and practice. As described before, the connection between knowledge and actual living situation is one of the most significant advantages of the in-group social learning. That this process is not automatic, and it requires a trained group leader was further noted by some of the participants.

“*Together with the group leader, we collected knowledge from our experiences, (…)” participant 271*.

At the same time, many participants observed that they were not only able to better learn from the experiences of others but also from professionals who were invited to the training.

“*Training was very pleasant but also very professional. The moderator was very helpful. Presented knowledge was very practical, and we could ask questions to the nurse, psychologist, and physiotherapist.” participant 16*.

As described in the method description and as could be seen from the experience exchange category, experience sharing also has a potential to create a positive and private atmosphere that enables further experience sharing, increases the likelihood of knowledge transfer (as described before) and helps people to understand others and relate to them.

“*I found that sharing experiences is very useful. Our group functioned great. I think we were very opened about our experiences* … *and one could freely share their emotions with others and would feel understood.” participant 412*.

These last two things (ability to understand others and relate to them) are very important advantages of the in-group social learning method since they decrease individuals' feelings of isolation in difficult situation (also mentioned under the benefit of social inclusion and social support in the next category). Furthermore, difficult situations that were once limiting can become an opportunity for personal development.

“*I found the training very useful and important because I could hear others' experiences, which made me feel I am not the only one in this kind of situation. Every person has different experiences, and I learned a lot from that*.” *participant 28*.

The potential of the in-group social learning for holistic personal development was also indicated by the subcategories in the inner strength category. Besides already mentioned increase in self-confidence, participants expressed the benefits of the training as stress relief and health strengthening.

Finally, as mentioned at the beginning, learning to relate to the experiences of others develops the human ability to experience empathy and solidarity. Analysis of item eight showed that comparing training participants to the representative sample of informal carers, training participants more frequently decided for (hypothetical) informal vs. formal care for themselves. This indicates, that training participants are experiencing informal care process more positively than carers who did not participate in the training, and so they would, despite all the burden of care they are familiar with, still rather receive care by a family member than a formal care employee—not to burden the family, but because they find the experience of giving and receiving reciprocally. This correlates well with the obtained qualitative data. Still, to better understand this process and possibilities it opens, further research is needed.

To conclude, the evaluation study shows that the in-group social learning method used for the informal carers training has great potential for quality care empowerment and development of new solidarity. The limitations of this study are foremost the lack of the usage of standardized instruments, which could offer more objective evaluation results, the lack of the information about the situation of each individual carer before the beginning of the training and absence of information on carer-care receiver relationship which could give us further insight into effectiveness of this method. Likewise, no information on group leader and the relationship between group leader and participants were systematically obtained. For further research, the potential positive and negative effect of the in-group social learning method-based informal carer's training on the care receivers' health, well-being, satisfaction with life or other emotional and personality aspects examination would be beneficial. Furthermore, the effectiveness of the in-group social learning method could also be compared to other similar methods.

## Conclusions

The review of presented in-group social learning method used for informal carers training has the following policy, practical and theoretical implications:

1. Informal carers provide 70–90% of all care in today's Europe. Training for carers who provide care to frail, chronically ill or disabled old persons, is prerequisite for sustainable and humane long-term care in the time of aging population. As shown by the evaluation study, in-group social learning method used for informal carers training is effective and has, therefore, the potential to be used by educational and long-term care systems.
2. The evaluation study shows that in-group social learning helps participants—informal carers—to gain needed skills and knowledge as well as develop personally. The connection between knowledge and actual living situation is achieved by experience sharing and processing and goes both ways—skills and knowledge needed by participants are simultaneously transferred from and to everyday practice. This method can be used by professionals as a bridge between their knowledge and the practical needs of the participants.
3. In-group social learning method used for the informal carers training develops carers' ability and motivation for caregiving, resulting in the gained capacity for the new solidarity. Therefore, we believe that the method of in-group social learning can valuably contribute to new findings and the development of new methods for the strengthening of post-traditional solidarity.

## Ethics Statement

This study was aprooved by Ethics Commision of Ministry of Health, Republic of Slovenia and in cooperation with Ageny for Public Health, Republic of Slovenia.

## Author Contributions

AR wrote a review of current international demographic and long-term care situations. JR provided the in-group social learning method description and examples of its implementation for informal carers training. AS provided the data organization, data analysis, and presented the results of the evaluation study. All authors contributed to the discussion and conclusion of the manuscript and its revision. All authors also read and approved the submitted manuscript.

### Conflict of Interest Statement

The authors declare that the research was conducted in the absence of any commercial or financial relationships that could be construed as a potential conflict of interest.
